# Thromboprophylaxis in multiple myeloma

**DOI:** 10.1016/j.rpth.2025.102685

**Published:** 2025-01-16

**Authors:** Laurent Frenzel

**Affiliations:** Haematology Unit, Necker Hospital, Imagine Institute, Paris, France

## 1 Introduction

The article titled “Multiple Myeloma: Retrospective Assessment of Routine Thromboprophylaxis and Utility of Thrombotic Risk Scores” [1] evaluates thrombotic risk and thromboprophylaxis in newly diagnosed multiple myeloma (NDMM) patients, focusing on the predictive capabilities of three well-established thrombotic risk scores (TRSs): IMPEDE-VTE, SAVED, and PRISM [2]. Thrombosis, particularly venous thromboembolism (VTE), is a well-known complication in multiple myeloma (MM), largely due to both the disease and its treatment, especially immunomodulatory drugs (IMiDs) like thalidomide and lenalidomide. The study’s findings are especially relevant for guiding thromboprophylaxis strategies in MM, particularly in resource-limited settings.

## 2 Risk of Thrombosis in MM and its impact on treatment

Thrombosis is one of the most common and severe complications in MM, with a direct impact on patient outcomes and treatment strategies. MM itself predisposes patients to a hypercoagulable state due to multiple factors, including the secretion of proinflammatory cytokines, increased platelet activation, and the use of thrombotic treatments like IMiDs and high-dose corticosteroids [3]. The introduction of IMiDs, such as thalidomide, lenalidomide, and pomalidomide, has revolutionized MM treatment, improving survival rates and response outcomes. However, this therapeutic gain comes with an elevated risk of VTE, which can significantly complicate patient management.

The incidence of VTE in MM patients can range between 10% and 20%, depending on risk factors, treatment regimens, and patient characteristics [4]. IMiDs combined with dexamethasone (Dex) have been particularly associated with higher thrombotic risks, especially when used in high doses [5]. This is consistent with the study’s reported overall VTE rate of 14.8% in patients receiving routine thromboprophylaxis with aspirin (ASA). Thrombosis not only increases morbidity and mortality in MM patients but also complicates their treatment, often requiring the temporary discontinuation of MM-specific therapies like IMiDs, which can negatively impact disease control.

The hypercoagulable state in MM is multifactorial. Tumor cells themselves can induce endothelial damage and increase the release of procoagulant factors such as tissue factor or extracellular microvesicles [6]. Moreover, monoclonal protein associated with MM can enhance blood viscosity and lead to clot formation. In addition, many MM patients present with comorbidities such as advanced age, cardiovascular disease, and immobility, all of which further elevate the risk of thrombotic events. Importantly, the presence of VTE in MM patients does not seem to directly affect overall survival according to some studies, but it significantly increases the risk of complications and negatively impacts the quality of life [7].

Furthermore, thrombotic events can lead physicians to modify or temporarily halt the administration of certain medications, especially IMiDs, until the thrombotic risk is mitigated [2]. Given that MM patients are often older and frail, this balancing act between treating the cancer and managing thrombosis becomes a critical aspect of care. In this study, the authors highlight that most thrombotic events occurred early, within the first few months of treatment initiation, underscoring the importance of early and adequate thromboprophylaxis. The challenge for clinicians is to provide optimal anticancer treatment while minimizing the risk of thrombosis.

## 3 Utility of Predictive TRSs in MM

To mitigate the risk of thrombosis, some risk stratification tools have been developed to guide clinicians in tailoring thromboprophylaxis strategies for MM patients. The IMPEDE-VTE [8], SAVED [9], and PRISM [10] scores were created to predict thrombotic risk in patients undergoing treatment with IMiDs, and the authors of this study evaluated their performance in a Latin American cohort of NDMM patients.

The IMPEDE-VTE score was developed to account for MM-specific factors, such as the use of IMiDs, the presence of central venous catheters, infection, and the use of Dex. In this study, the authors found that the IMPEDE-VTE score performed better than both the SAVED and PRISM scores in stratifying patients according to their thrombotic risk. Specifically, patients classified as intermediate-risk by the IMPEDE-VTE score had a 7.14% incidence of VTE, while those in the high-risk group had a 13.2% incidence. However, these results were not statistically significant, likely due to the small sample size.

The performance of the SAVED and PRISM scores was less promising, with most patients being classified as high-risk, limiting their ability to stratify patients effectively. The SAVED score was initially developed for predicting VTE risk in patients treated with IMiDs, focusing on patient characteristics such as age, recent surgery or Dex uses, and obesity [11]. However, in this study, it failed to differentiate between low- and high-risk patients, potentially due to the different population demographics. The PRISM score, which incorporates cytogenetic abnormalities, also performed poorly in this cohort.

These findings are consistent with other studies that have shown the limited utility of the SAVED and PRISM scores in predicting VTE risk in non-Western populations. For example, some studies highlighted that these risk models may not be universally applicable due to differences in genetic and environmental factors [12]. The authors’ analysis enhances the need for further refinement and validation of TRS models to improve their predictive accuracy in real-world settings, particularly in resource-limited environments like Latin America. One potential strategy to optimize the management of thrombotic risk could be to adapt thromboprophylaxis not generally based on risk scores, but rather directly according to the treatment, as proposed by the IFM group [2]. Patients treated with an IMiD-Dex–based regimen, which represents the majority of first-line therapies [13], immediately require antithrombotic prophylaxis without the need for score calculation. However, this strategy is only valid with effective and well-tolerated antithrombotic treatments.

## 4 Thromboprophylaxis Strategies in MM: Is this the End of ASA?

Despite the increased risk of thrombosis in MM patients, the optimal choice of thromboprophylaxis remains a topic of debate. ASA is frequently used for thromboprophylaxis in MM due to its low cost and ease of administration, especially in resource-limited settings [14]. However, recent data, including those presented in this study, question the efficacy of ASA in high-risk patients, particularly those receiving IMiDs [2].

The authors report a VTE incidence of 14.8% in patients receiving ASA, a finding that is consistent with previous studies showing that ASA may not provide adequate protection in MM patients at high thrombotic risk. Additionally, the risk of major bleeding associated with ASA, as observed in 4.8% of patients in this study, further complicates its use.

Given these limitations, recent guidelines have shifted toward recommending direct oral anticoagulants (DOACs) or low-molecular-weight heparin as first-line thromboprophylaxis agents for high-risk MM patients. DOACs, such as apixaban and rivaroxaban, have been shown to provide superior protection against VTE with a lower risk of bleeding compared to ASA [15]. Others studies tend to demonstrate the efficacy and safety of DOACs in MM patients receiving IMiD-based therapies, and these agents are now considered a preferred option for high-risk patients [[16], [17], [18]]. The authors of this study report that only 2 patients received apixaban from the start, while 4 others transitioned from ASA to apixaban during follow-up.

## 5 Conclusion

This study highlights the complexity of managing thrombotic risk in NDMM patients, particularly those receiving IMiD-based therapies. While predictive TRSs like IMPEDE-VTE, SAVED, and PRISM have been developed to aid in stratifying patients’ risks, this study demonstrates the limited utility of some of these tools, particularly in non-Western populations. The results reinforce the need for tailored approaches to thromboprophylaxis, especially in resource-limited settings where ASA remains widely used despite its questionable efficacy in high-risk patients. The study suggests a shift toward more effective antithrombotic agents such as DOACs and low-molecular-weight heparin, especially for patients at higher thrombotic risk, to ensure optimal protection against VTE without compromising patient safety through excessive bleeding. As evidence grows in support of these newer therapies, clinical practice must evolve to ensure that MM treatment strategies effectively balance cancer control and thrombosis prevention, improving patient outcomes and quality of life.
